# Dietary Diversity Is Not Associated with Haematological Status of Pregnant Women Resident in Rural Areas of Northern Ghana

**DOI:** 10.1155/2017/8497892

**Published:** 2017-01-11

**Authors:** Mahama Saaka, Jeremiah Oladele, Asamoah Larbi, Irmgard Hoeschle-Zeledon

**Affiliations:** ^1^School of Allied Health Sciences, University for Development Studies, P.O. Box 1883, Tamale, Ghana; ^2^International Institute of Tropical Agriculture (IITA), P.O. Box 6, Tamale, Ghana; ^3^International Institute of Tropical Agriculture (IITA), Oyo Road, PMB 5320, Ibadan, Nigeria

## Abstract

*Background*. Information regarding how dietary diversity is related to haematological status of the pregnant women in rural areas of Northern Ghana is limited. This study therefore evaluated maternal dietary intake and how it relates to the nutritional status of pregnant women belonging to different socioeconomic conditions in Northern Ghana.* Methods*. This study was cross-sectional in design involving 400 pregnant women. Midupper arm circumference (MUAC) and anaemia status were used to assess the nutritional status of pregnant women.* Results*. The mean dietary diversity score (DDS) of the study population from ten food groups was 4.2 ± 1.5 (95% CI: 4.08 to 4.37). Of the 400 women, 46.1% (95% CI: 40.0 to 52.2) met the new minimum dietary diversity for women (MDD-W). The mean haemoglobin concentration among the pregnant women studied was 10.1 g/dl ± 1.40 (95% CI: 9.8 to 10.3). The independent predictors of haemoglobin concentration were maternal educational attainment, gestational age, frequency of antenatal care (ANC) attendance, number of under-five children in the household, size of MUAC, and maternal height.* Conclusions*. Irrespective of the socioeconomic status, women minimum dietary diversity (MDD-W) was not associated with anaemia among pregnant women resident in the rural areas of Northern Ghana.

## 1. Introduction

Pregnant women in developing countries including Ghana enter gestation with depleted or low body iron stores which might make them prone to developing iron deficiency anaemia (IDA) [1]. Such women are most vulnerable because of the increased metabolic demands including a growing placenta, foetus, and maternal tissues, coupled with poor diets [2]. This vulnerability often leads to adverse effects such as premature delivery, intrauterine growth retardation, increased risk of malnutrition, mortality, and morbidity on both mother and growing foetus [1, 3–5]. During pregnancy, dietary energy and nutrient requirements are generally increased to support increased maternal metabolism, blood volume, and red cell mass expansion, and the delivery of nutrients to the foetus.

Several studies have shown a lack of diversity among poor populations, whose diets are predominantly carbohydrates with little or no animal products, fruits, and vegetables [2, 5]. Multicountry studies have provided empirical evidence that dietary diversity scores are reliable measures for micro- and macronutrient adequacy for women of reproductive ages [6]. So a diverse diet of a pregnant woman is expected to improve her nutritional profile and thus reduce the risk of maternal and child mortality and morbidity. If the diet lacks diversity, it will be deficient in essential nutrients such as iron which is required for blood formation.

It is well documented that adequate nutritional status of women especially during pregnancy is crucial for child survival because an undernourished mother is more likely to deliver an infant with low birth weight, significantly increasing its risk of dying [7–12]. A pregnant woman whose diet lacks diversity is most likely to be deficient in essential nutrients, thereby depriving the foetus of the nutrition it requires to have a healthy growth [13–15].

Though dietary diversity is a recognized indicator of diet quality, there is limited knowledge on how it affects the nutrition status of pregnant women in rural areas of Northern Ghana. This study therefore sought to assess dietary diversity and how it relates to the nutritional status of pregnant women belonging to different socioeconomic conditions in Northern Ghana.

## 2. Methods

### 2.1. Study Area

The study covered 25 communities located in five districts of Northern Ghana. The International Institute of Tropical Agriculture (IITA) is currently operating in these districts, namely, Nadowli, Wa West in the Upper West Region (UWR), Tolon and Savelugu in the Northern Region (NR), and Kassena-Nankana in the Upper East Region (UER).

The study area is characterized by high poverty and recurrent droughts and floods which predispose communities to increased vulnerability to food insecurity and malnutrition. The Ghana Living Standards Survey Round 6 Report showed that the three study regions have high proportions of households in the lowest quintile than in the highest quintile. The UWR has the highest proportion of households in the lowest quintile (55.7%) and the NR recorded the lowest proportion in the highest quintile (10.2%) [16]. These are indication that poverty is more prevalent in the three northern regions, particularly in the Upper West Region.

The three regions share some boundaries with each other. The UER shares its northern boundary with Burkina Faso and its eastern boundary with the Republic of Togo. The UWR on the other hand has a northern boundary with Burkina Faso and a western boundary with La Cote d'Ivoire. The UER and UWR share their southern boundaries with the NR which also has La Cote d'Ivoire to the west and Togo to the east.

The majority of the people have agriculture as their main occupation whilst some are involved in trading. The main staple foods including maize, sorghum, millet, and yam are usually harvested from October through December. Although the food security situation is usually good during the harvesting time, child care tends to suffer because of lack of time on the part of rural mothers. A high proportion of rural women work daily away from home and therefore frequently face challenges to the care of children.

The rainfall pattern is unimodal and the period is usually short and lasts from May to August, followed by a long dry season (September–April) with dry harmattan winds.

### 2.2. Study Design, Population, and Sampling

This study was an analytical cross-sectional survey involving 400 pregnant women in different stages of gestation, conducted in 5 districts of Northern Ghana. All the IITA five communities in each district were included in the study. All women independent of their stage of pregnancy were asked to participate in the study if they met the inclusion criteria. The inclusion criteria were being pregnant but not seriously ill. Also, pregnant women who could not hear and/or speak were excluded from the study.

The basic primary sampling unit was the household in which there was a pregnant woman. With the assistance of community health volunteers, a complete list of all households was compiled and were serially numbered in each community. Systematic random sampling was then used in selecting study households. To get the sampling interval, the total number of households in a cluster was divided by the cluster size of 16. The first household was randomly selected by picking any number within the sample interval. Subsequent selections were made by adding the sampling interval to the selected number in order to locate the next household to visit. If the selected household does not have a target respondent, then next household was selected using the systematic sampling procedure.

The sample size was determined on the assumption that 50% of the pregnant mothers are anaemic with 5% marginal error and 95% confidence interval (CI) and a nonresponse rate of 5%. Based on this assumption, the actual sample size for the study was determined using the formula for one-point sample estimation:

(1) $$\begin{array}{c} n=\frac{t^{2}\times p\left(1-p\right)}{m^{2}}, \end{array}$$

where *n* is the required sample size, *t* is the confidence level at 95% (standard value of 1.96), *p* is the estimated prevalence of anaemia in the domain area (50.0%), and *m* is the margin of error at 5% (standard value of 0.05). This yielded a minimum sample size of 400.

### 2.3. Data Collection

Information was obtained through a face-to-face interview with mothers in their homes. The primary dependent variable was anaemia status of pregnant women. The main independent explanatory variable was dietary quality as measured by individual dietary diversity scores. The covariate variables included gestational age, marital status, maternal age, height, educational level, occupation, religion, household wealth, number of under-fives in household, parity, birth interval, antenatal care (ANC) during current pregnancy, and midupper arm circumference (MUAC).

### 2.4. Assessment of Dietary Diversity

Overall dietary quality was assessed using the individual dietary diversity since it has been shown to indicate adequate nutrient intake [5, 17] and can be used as a proxy indicator for measuring nutrient adequacy among pregnant females [1]. Dietary assessment was made through administration of the 24-hour food-frequency questionnaire (FFQ).

Women's dietary diversity scores (WDDSs) which are based on a 24-hour dietary recall period and the number of food groups consumed [18] were applied to characterize the average usual dietary intake of pregnant women in the study area. The food groups used were all starchy staple foods, beans, peas, nuts, seeds, dairy, flesh foods, eggs, vitamin A-rich dark green leafy vegetables, other vitamin A-rich vegetables and fruits, other vegetables, and other fruits.

Women having a diversity score of less than 5 were classified as having low dietary diversity and scores of 5–10 were classified in the high dietary diversity scores.

### 2.5. Nutritional Status Assessment

Midupper arm circumference (MUAC) and haemoglobin levels were used to assess the nutritional status of the pregnant women. MUAC was used as a proxy for body weight, since it is not affected by gestational age [19]. MUAC was measured to the nearest 0.1 cm, and values below 25.0 cm were classified in the analyses as an indicator of low body weight.

There is currently no internationally agreed MUAC cutoffs. A recent multicountry study concluded that it may be difficult to recommend a MUAC cutoff that would be suitably discriminatory in all settings [20].

### 2.6. Haemoglobin Level Determination

Haemoglobin levels were determined by using a portable HemoCue 301 photometer. Trained laboratory technicians drew capillary blood samples from the finger prick with a lancet after taking all aseptic precautions. The first drop of blood was wiped away using alcohol sterile wipes, and the next drop was placed into the HemoCue curvette for immediate testing of haemoglobin.

According to WHO, anaemia is defined as presence of haemoglobin level of less than 11 g/dL in pregnant women [21]. Anaemia was further classified as mild (9.0–10.9 g/dL), moderate (7.0–8.9 g/dL), or severe (<7.0 g/dL). Anaemia is said to be a severe public health problem when its prevalence is 40% or more in any group (all types of anaemia) or when severe anaemia (haemoglobin < 7 g/dL) exceeds 2% [22].

### 2.7. Determination of Household Economic Status

A household wealth index based on household assets and housing quality was used as a proxy indicator for socioeconomic status (SES) of households. Principal Component Analysis (PCA) was used to determine household wealth index from information collected on housing quality (floor, walls, and roof material), source of drinking water, type of toilet facility, the presence of electricity, type of cooking fuel, and ownership of modern household durable goods and livestock (e.g., bicycle, television, radio, motorcycle, sewing machine, telephone, cars, refrigerator, mattress, bed, computer, and mobile phone) [23–26].

These facilities or durable goods are often regarded as modern goods that have been shown to reflect household wealth. A household of zero index score, for example, means that household had not a single modern good. The scores were thus added up to give the proxy household wealth index. The median of the total score was the cutoff point used to define low and high household wealth index.

### 2.8. Data Processing and Analysis

Data were analyzed using SPSS version 20 statistical software. Analysis of variance (ANOVA) was used to compare group mean differences. Association between anaemia and some risk factors in pregnancy was tested using chi-square and multivariable analysis of risk factors. Variables with *p* value less than 0.1 in bivariate analysis were entered to multivariable linear regression model. Differences and associations were considered statistically significant at *p* < 0.05. Multicollinearity was investigated by using the variance inflation factor (VIF). A VIF (the reciprocal of the tolerance statistics) of greater than 5 is generally considered evidence of multicollinearity.

### 2.9. Ethical Considerations

The School of Allied Health Sciences, University for Development Studies, approved the study protocol. Ethical clearance was obtained from the Institutional Review Board (IRB) of the Tamale Teaching Hospital (Ref number TTH/10/11/15/01). Participation in the study was voluntary and no incentives were provided.

## 3. Results

### 3.1. Sociodemographic Characteristics of the Sample

A total of 400 pregnant women were approached and all of them consented and accepted to participate in the study, thus giving a response rate of 100%. The mean age of mothers was 26.2 ± 6.4 years which range from 15 to 48 years. Majority (54.0%) of the respondents were Muslims. Majority (85.5%) of the respondents were married and (51.8%) of the mothers had no formal education at all. Farming and petty trading were common among the mothers (Table 1).

### 3.2. Maternal Dietary Diversity during Pregnancy

The mean dietary diversity score (DDS) of study population from ten food groups was 4.2 ± 1.5 (95% CI: 4.08 to 4.37). Of the 400 women, 46.1% (95% CI: 40.0 to 52.2) met the MDD-W.

### 3.3. Nutritional and Anaemia Status of Pregnant Women

Based on MUAC cutoffs, the overall prevalence of underweight (MUAC < 25.0 cm) was 28.8% (95% CI: 24.6 to 33.4), whilst 71.2% of the respondents were classified as normal (MUAC ≥ 25.0 cm).

The mean haemoglobin concentration among the pregnant women studied was 10.1 g/dl ± 1.40 (95% CI: 9.8 to 10.3), and an overall prevalence of anaemia (haemoglobin level < 11 g/dl) was 70.0% (CI: 60.5 to 78.0). In terms of severity, mild anaemia was 46.8% (95% CI: 37.9 to 56.0), moderate anaemia was 20.2% (CI: 15.8 to 25.5), and severe anaemia was 3.0% (CI: 1.4 to 6.0). The prevalence of anaemia was the highest in the Northern Region.

### 3.4. Analysis of Haemoglobin Levels Based on the Type of Food Groups Consumed among Pregnant Women

Analysis of haemoglobin cutoffs levels based on the type of foods consumed is shown in Table 2. Generally, consumption of food groups in the past 24 hours prior to the study was not associated with haemoglobin concentration levels.

### 3.5. Relationship between Women Minimum Dietary Diversity and Anaemia

The relationship between women minimum dietary diversity (MDD-W) and anaemia was investigated. Correlation analysis showed a positive association between household wealth index and dietary diversity score (DDS) (*r* = 0.2, *p* < 0.001) but DDS was unrelated to Hb irrespective of the socioeconomic class. For the whole sample, DDS did not have an association with haemoglobin status of pregnant women (*r* = −0.001, *p* = 0.9). Similarly, the prevalence of anaemia was not different among women of low and high MDD-W (36.2 versus 33.8%) (*χ*^2^ = 1.9; *p* = 0.17). This association existed, irrespective of the socioeconomic status.

### 3.6. Factors Associated with Anaemia

Bivariate analyses were performed to assess association of sociodemographic and other factors with anaemia (Table 3). The overall prevalence of anaemia fell with gestational age. Both educational level and household wealth index were negatively associated with anaemia. Pregnant women in the Upper West Region had a lower prevalence of anaemia than Northern and Upper East Regions. The bivariate analysis showed that pregnant women with a midupper arm circumference (MUAC) less than 25 cm were more likely to be anaemic, compared to MUAC ≥ 25.0 cm (79.0% versus 58.9%).

A multiple linear regression analysis was performed to identify the predictors of haemoglobin concentration. Potential explanatory variables that were tested but found insignificant were dietary diversity, age of woman, and reported episodes of malarial infections. Region of residence correlated with a number of the explanatory variables and was therefore removed from the model.

The significant independent determinants of Hb were maternal educational attainment, gestational age, frequency of ANC attendance, number of under-five children in the household, size of MUAC, and maternal height. The set of independent variables could explain 11.3% of the variance in haemoglobin concentration (Adjusted *R* Square = 0.113).

Considering the beta coefficients (*β*), women who attended ANC at least four times had mean Hb concentration which was 0.147 standardized units significantly higher than women who attended less than four times. There was an inverse relationship between the prevalence of anaemia and the level of education of the women. Mean Hb of women whose educational level was at least senior high school was significantly higher by 0.122 units. Women of higher MUAC had higher Hb (beta coefficient = 0.216, *p* < 0.001). On the average, a unit increase in gestational age was associated with reduced mean Hb of 0.172 standardized units. A 1 cm increase in maternal height was associated with an increase of 0.14 units in haemoglobin concentration (*β* = 0.141, *p* = 0.006) (Table 4).

## 4. Discussion

The dietary intake of a woman during pregnancy is important because suboptimal diet negatively impacts the health of the mother, the foetus, and the newborn. This study therefore sought to assess maternal dietary intake and how this relates to the haematological status of pregnant women belonging to different socioeconomic conditions in Northern Ghana.

The risk factors of anaemia during pregnancy are multifactorial and the relative contribution of each of these factors varies greatly by geographical location, season, and dietary practice [27]. Whilst controlling for potential confounding factors in multivariable analysis, haematological status of pregnant women was not associated with maternal dietary diversity score (DDS). The significant independent determinants of Hb were maternal educational attainment, gestational age, frequency of ANC attendance, number of under-five children in the household, size of MUAC, and increased maternal height.

In bivariate analysis, some potential explanatory variables including household wealth index, self-reported episodes of malarial infection, and maternal age were tested but found insignificant.

### 4.1. Prevalence of Anaemia

The prevalence of anaemia among pregnant women was 70.0% and this can be classified as a severe public health problem according to WHO [22]. The prevalence is higher than the 51.9–59.6% estimated prevalence of anaemia in pregnancy in Africa [28]. The finding is also higher than that from the 2014 Ghana Demographic and Health Survey (GDHS) in which, overall, 44.6% of pregnant women in Ghana suffered from some degree of anaemia [29].

### 4.2. Dietary Diversity and Haematological Status

The results of this study showed that whilst controlling for potential confounding factors in multivariable analysis, haematological status of pregnant women was not associated with dietary diversity score (DDS) in rural settings. This finding collaborates that of a study carried out in Pakistan [30]. However, in an earlier study conducted in an urban setting of Northern Ghana, dietary diversity of pregnant women had positive effect on Hb in the third trimester [31]. In the present study, all pregnant women irrespective of the gestational age were included in the analysis.

The relationship between dietary diversity and anaemia remains inconclusive. Some other studies have reported a lack of association between dietary diversity and the prevalence of anaemia, especially in environments where many other factors other than diet exposure can increase the risk of getting anaemia [32, 33]. It has also been reported that some pregnant women do restrict dietary intake in order to have smaller babies and therefore easier deliveries [34, 35]. In Ethiopia, women with restrictive dietary habits were reported to have 39% higher risk of anaemia compared to those without restrictive dietary behaviour [36] where maternal dietary diversity was protective of pregnancy anaemia [37–39].

Dietary diversity is considered to be a key indicator for assessing the access, utilization, and quality of diet of individuals or household [40]. Though high quality diet can protect against anaemia, some eating patterns or habits may predispose individuals to a higher risk for developing anaemia. For example, high fibre diets can inhibit the absorption of iron; low fat diets can equally inhibit iron absorption since fat is needed for iron absorption, high tea, and coffee consumption but without vitamin C intake it also inhibits iron absorption. Individual dietary diversity scores have been shown to indicate adequate nutrient intake and it can be used as a proxy indicator for measuring nutrient adequacy among pregnant females [41].

This notwithstanding women's dietary behaviours and intake during pregnancy are strongly influenced by different cultural practices, myths, and taboos [42, 43].

The aetiology of anaemia in pregnancy does not seem to be the same in every geographical area and season. Whereas, in Malawi, iron deficiency was an important contributor to anaemia in pregnancy, Saaka et al. [44] reported that iron deficiency did not appear to be a major risk factor for anaemia among pregnant women in the Agogo district of Ghana. Additionally, diet is only one of the protective factors against anaemia and dietary diversity may not be the most pressing constraint. The apparent lack of association in the present study may also be due to the fact that there was very little variation with respect to dietary diversity among the study participants.

### 4.3. ANC Attendance and Anaemia

Our results showed that prevalence of anaemia among women who attended antenatal care at least four times was significantly lower and they were less likely of becoming anaemic. The positive contribution of early initiation of ANC attendance to haematological status is probably due to the benefits associated with ANC. For example, women who seek ANC frequently are more likely to benefit from prophylactic measures against malarial infection, iron, and folic acid supplementation and that of nutrition and health education. The finding supports previous studies which have reported an association between infrequent prenatal consultations and the presence of anaemia [45–48]. Similarly, the number of antenatal care visits was a strong predictor of anaemia among pregnant women in Westmoreland, Jamaica [48].

There is an increased foetal demand for haematopoietic factors as pregnancy progresses [49, 50] and so women who will not avail themselves to health services early enough may suffer the consequences of increased demand for nutrients. Such women are also more unlikely to take advantage of accessing health services to treat any underlying maternal diseases and untreated anaemia in early pregnancy that are likely to worsen in the course of pregnancy. In this study sample, less than 16% reported for ANC in their first trimester and so many of the women did not present for antenatal care until being late in the second trimester of pregnancy. Pregnant women in the study area should be educated to report early for ANC.

Though iron supplementation is key intervention for pregnant women in the study area, late and infrequent ANC attendance will deny these women the benefits of such an intervention. Also, nonadherence to the recommended iron supplementation could adversely affect their iron status but our study did not assess this factor.

### 4.4. Anaemia and Maternal Education

Low maternal educational level significantly predicted anaemia in this study. Women who had higher education level were less likely to be anaemic, perhaps because better educated mothers were more likely to adopt healthier feeding habits [19, 30]. This could also be explained partly by the fact that women with lower educational level may find it difficult securing better jobs and therefore have less economic power. Education may also enable women to make independent decisions that allow them to have greater access to household resources that are important to nutritional status [51]. Our results further give support to the call to invest in girl child education as a way to promoting the general wellbeing of future mothers [48, 52].

### 4.5. Maternal Height and Risk of Anaemia

In adjusted models, a 1 cm increase in maternal height was associated with a decreased risk of anaemia. This means maternal height and haemoglobin concentrations were directly associated. This association has been reported in earlier studies [53]. Adult height reflects a mother's health status that has been acquired through various life experiences including the social and environmental exposures [54].

### 4.6. Household Size and Risk of Anaemia

In this study sample, there was an inverse relationship between the prevalence of maternal anaemia and the number of under-five children in the household. This is consistent with the findings of similar studies conducted in Kuwait where anaemia was positively associated with birth order and in Amazonia where high maternal parity is positively associated with anaemia [55, 56]. This relationship may be explained by the fact that a woman who gives frequent birth is more likely to deplete her iron stores.

### 4.7. Maternal Nutritional Status and Anaemia

Multivariable regression analysis showed that pregnant women of higher midupper arm circumference (MUAC) ≥25.0 cm had significantly higher haemoglobin concentrations. A study in rural northern Tanzania had earlier reported that an arm circumference less than 25 cm was associated with anaemia [57]. Though MUAC is unaffected by gestational age [19], prolonged infections can adversely affect the size of it as a result of their consequences leading to both anaemia and wasting and therefore an apparent association between the arm circumference and anaemia [57].

### 4.8. Limitation of the Study

Dietary diversity was assessed based on responses obtained from participants (e.g., dietary recall) during the pregnancy and this depended on memory and their ability to recall accurately. Recall bias could not be ruled out completely. The 24-hour dietary recall may not truly represent the usual intake. However, methods used in assessing dietary diversity are useful for ranking individuals but do not necessarily permit exact assessments of absolute nutrient intake.

The study also relied partly on secondary data about participants recorded by health professionals during the pregnancy. Therefore, any error in measurements, readings, or recordings of these parameters and indices will be reflected in the results. However, with the level of professionalism of health workers in the institutions involved in the study, this is expected to be minimal. The cross-sectional study design used to collect data also makes it difficult to demonstrate cause-and-effect relationships.

It is also worth mentioning that it was not possible to obtain haematimetric and biochemical indicators necessary for the diagnosis and estimation of the magnitude of iron deficiency by virtue of the available haematological evaluation technique that was used in the study.

## 5. Conclusions

Whilst controlling for potential confounding factors in multivariable analysis, haematological status of pregnant women was not associated with maternal dietary diversity score (DDS) irrespective of the socioeconomic status. The study findings suggest that anaemia was higher with increased gestational age and lower educational level. This implies the need to target interventions to these vulnerable groups of women. Furthermore, making early and timely visits to the antenatal clinic would help to lower the prevalence of anaemia among pregnant women in Northern Ghana.

## Figures and Tables

**Table 1:** Sample characteristics (*N* = 400).

Variable	Frequency (*n*)	Percentage (%)
Age groupings (years)		
15–24	176	46.5
25–34	180	44.6
35–49	44	8.8
Religion		
Christian	176	44.0
Muslim	216	54.0
Traditionalist	8	2.0
Classification of occupation		
Trader	98	24.5
Farmer	123	30.8
Civil servant	2	0.5
Service worker	67	16.8
Education/teacher	5	1.3
Health worker	3	0.8
Nothing	102	25.5
Ethnicity		
Dagomba	119	29.8
Kassena	71	17.8
Dagaba	110	27.5
Kukomba	2	0.5
Frafra	14	3.5
Wala	55	13.8
Fulani	2	0.5
Dorimor	27	6.8
Education level of mother		
None	207	51.8
Low (primary and junior high)	157	39.3
High (≥senior high)	36	9.0
Marital status		
Single	8	2.0
Married	342	85.5
Widowed	5	1.3
Cohabiting	33	8.3
Separated	12	3.0

**(a):** 

Indicator	*N*	Mean Hb	Std. deviation	95% confidence interval for mean		Test statistic
				Lower bound	Upper bound	
Women's minimum dietary diversity						
<5	227	10.37	1.48	10.18	10.56	*F*(1,398) = 0.8, *p* = 0.37
≥5	173	10.24	1.37	10.03	10.44	
Consumption of staple food (cereals, roots, and tubers)						
No	33	10.50	1.40	10.005	10.99	*F*(1,398) = 0.6, *p* = 0.43
Yes	367	10.30	1.44	10.15	10.44	
Consumption of vitamin A-rich dark green leafy vegetables						
No	112	10.13	1.56	9.84	10.43	*F*(1,398) = 2.4, *p* = 0.12
Yes	288	10.38	1.38	10.22	10.54	
Other vitamin A-rich vegetables and fruits						
No	214	10.26	1.51	10.06	10.46	*F*(1,398) = 0.6, *p* = 0.45
Yes	186	10.37	1.35	10.18	10.57	
Other vegetables						
No	331	10.21	1.44	10.06	10.37	*F*(1,398) = 19.3, *p* = 0.002
Yes	69	10.79	1.30	10.48	11.11	
Other fruits						
No	369	10.30	1.44	10.15	10.44	*F*(1,398) = 0.5, *p* = 0.5
Yes	31	10.49	1.41	9.98	11.01	

**(b):** 

Indicator	*N*	Mean Hb	Std. deviation	95% confidence interval for mean		Test statistic
				Lower bound	Upper bound	
Flesh food (e.g., red meats, fish)						
No	101	10.31	1.45	10.03	10.60	*F*(1,398) = 0.0, *p* = 1.0
Yes	299	10.31	1.43	10.15	10.47	
Eggs						
No	357	10.30	1.42	10.15	10.45	*F*(1,398) = 0.4, *p* = 0.5
Yes	42	10.45	1.55	9.97	10.94	
Seeds and nuts						
No	166	10.51	1.31	10.31	10.71	*F*(1,398) = 5.5, *p* = 0.02
Yes	234	10.17	1.50	9.98	10.36	
Beans						
No	294	10.39	1.41	10.23	10.55	*F*(1,398) = 3.3, *p* = 0.07
Yes	104	10.09	1.50	9.80	10.39	
Dairy products						
No	330	10.36	1.46	10.20	10.52	*F*(1,398) = 1.9, *p* = 0.17
Yes	69	10.10	1.30	9.79	10.41	

**Table 3:** Bivariate analyses of factors associated with anaemia of pregnant women (*n* = 400).

Characteristic	*N*	Anaemia		Test statistic
		No *n* (%)	Yes *n* (%)	
Household wealth index				
Low	223	70 (31.4)	153 (68.6)	*χ* ^2^ = 5.2, *p* = 0.02
High	177	75 (42.4)	102 (57.6)	
Midupper arm circumference				
<25 cm	100	21 (21.0)	79 (79.0)	*χ* ^2^ = 10.2, *p* < 0.001
≥25.0 cm	297	122 (41.1)	175 (58.9)	
Educational level				
None	207	61 (29.5)	146 (70.5)	*χ* ^2^ = 13.1, *p* < 0.006
Low (primary and junior high)	157	65 (41.4)	92 (58.6)	
High (at least senior high)	36	19 (52.8)	17 (47.2)	
Gestational age				
First trimester	63	35 (55.6)	28 (44.4)	*χ* ^2^ = 12.0, *p* = 0.002
Second trimester	143	48 (33.6)	95 (66.4)	
Third trimester	193	62 (32.1)	131 (67.9)	
U-fives in household				
None	100	46 (46.0)	54 (54.0)	*χ* ^2^ = 7.9, *p* = 0.02
1-2	203	73 (36.0)	130 (64.0)	
At least 3	97	26 (26.8)	71 (73.2)	
Region of residence				
Northern	126	24 (19.0)	102 (81.0)	*χ* ^2^ = 31.1, *p* < 0.001
Upper East	84	27 (32.1)	57 (67.9)	
Upper West	190	94 (49.5)	96 (50.5)	

**Table 4:** Determinants of haemoglobin concentration (g/dl) of pregnant women (multiple linear regression analysis).

Model	Standardized coefficients	Sig.	95.0% confidence interval for (*β*)		Collinearity statistics	
	Beta		Lower bound	Upper bound	Tolerance	VIF
(Constant)		0.001	2.27	8.12		
Educational level	0.122	0.013	0.06	0.48	0.96	1.05
Frequency of ANC visits	0.147	0.008	0.120	0.784	0.75	1.33
Gestational age of woman	−0.172	0.002	−0.55	−0.12	0.74	1.35
Under-five children in household	−0.117	0.017	−0.37	−0.04	0.95	1.05
MUAC of mother	0.216	<0.001	0.05	0.12	0.87	1.15
Height of mother	0.141	0.006	0.006	0.04	0.89	1.12
